# Evaluation of a 3‐dimensional ultrasound device for noninvasive measurement of urinary bladder volume in dogs

**DOI:** 10.1111/jvim.15811

**Published:** 2020-05-28

**Authors:** Matthew R. DiFazio, Justin D. Thomason, Natalia Cernicchiaro, David Biller, Sasha Thomason, Paxton Harness

**Affiliations:** ^1^ Department of Clinical Sciences: Radiology, Kansas State University College of Veterinary Medicine Manhattan Kansas USA; ^2^ Department of Clinical Sciences: Cardiology, Kansas State University College of Veterinary Medicine Manhattan Kansas USA; ^3^ Department of Diagnostic Medicine/Pathobiology, Kansas State University College of Veterinary Medicine Manhattan Kansas USA; ^4^ Department of Clinical Sciences: Small Animal Emergency Medicine, Kansas State University College of Veterinary Medicine Manhattan Kansas USA; ^5^ Pax Imaging PLLC Hayden Idaho USA

**Keywords:** distension, dysuria, micturition, retention, urinary, volumetry

## Abstract

**Background:**

The BladderScan Prime Plus (BPP; Verathon, Bothell, Washington) is an application‐specific, three‐dimensional ultrasound device used for human, point‐of‐care volumetry of the urinary bladder.

**Objective:**

To estimate the BPP's accuracy, repeatability, and optimized settings for assessing urinary bladder volumes in dogs, a variable utilized in assessing micturition disorders.

**Animals:**

Twenty‐four, client‐owned, healthy, male dogs presenting for routine examination.

**Methods:**

Prospective examinations were conducted by an experienced ultrasonographer and a novice, selecting the BPP's “man” or “child” setting, and were compared to urine volume obtained by catheterization.

**Results:**

Mean urine volume significantly varied by operator (*P* = .05), device setting (*P* < .001), and weight (*P* = .01); the “man” setting produced mean volumes nearer to catheterized volumes. The mean difference between BPP's “man” setting and catheterized volume was 0.88 mL, with maximal positive and negative disagreement of +23.2 mL to −55.3 mL (SD 19.0). Percent disagreement between BPP and catheterized volumes demonstrated a mean of −4.5%, with maximal positive and negative disagreement of +58.1% to −74.1% (SD 34.9). The experienced operator recorded volumes significantly (*P* = .05) higher than the novice, with difference in means of 3.2 mL. In dogs weighing >5.5 kg (n = 18/24), mean difference between BPP's “man” setting and catheterized measurements, regardless of operator, was not significant.

**Conclusions:**

Although small magnitude interuser variability is present in BPP examinations, the device provides accurate, though imprecise quantification of bladder volume in canids weighing >5.5 kg.

## 1 INTRODUCTION

Urinary retention disorders resulting from obstructive and functional abnormalities are common in small animal practice, and diagnosis of these disorders can require careful consideration of history, monitoring of urinary output, repeated bladder palpations, or ultrasonography to fully characterize.^1^, ^2^, ^3^, ^4^, ^5^, ^6^, ^7^, ^8^, ^9^, ^10^, ^11^, ^12^, ^13^, ^14^

The BladderScan Prime Plus (BPP; Verathon, Bothell, Washington) is a portable three‐dimensional (3D) ultrasound instrument that utilizes a proprietary convolutional neural network trained on a database of >38 000, radiologist‐reviewed, human clinical images to recognize bladder boundaries and report urine volume.^15^ Its design is intended to provide quick, accurate, and noninvasive measurement capability. Manufacturer‐proposed use cases include the diagnosis of common urological conditions, assessment of urine retention, prevention of unnecessary catheterization, and reduction in the rates of catheter‐associated urinary tract infections.^15^ The device produces a real‐time, B‐mode sonogram that enables the user to center the urinary bladder in the probe's field, while providing a constantly adjusting overlay that depicts the bladder boundaries as perceived by the device. Once the user is satisfied with positioning, the probe is held stationary and the device mechanically rotates an internal transducer to acquire data from 12, radially oriented planes which bisect the bladder. The device assembles a 3D data set, assesses bladder volume algorithmically, and reports a numerical result on‐screen within a few seconds. At the time of publication, the manufacturer claims accuracy to within ±7.5% on volumes over 100 mL and ±7.5 mL on volumes less than 100 mL in humans.^15^

Clinical validation in humans found the BPP to underperform relative to the authors' arbitrary limit of ±5% of catheterized urine volume for all examinations, with an average underestimation of −4.0%, which falls within manufacturer claims.^15^, ^16^ These results cannot reflexively be generalized to veterinary species, because of interspecies differences in bladder morphology and positioning within the abdomen. The objective of this study was to estimate the BPP's accuracy, repeatability, and optimized settings for assessing urinary bladder volumes in dogs, in comparison to urine volume obtained by catheterization, under differing operator experience, device settings, and size of the dogs as assessed by weight. This represents the initial step in determining the applicability of a device validated for human use, for use in canids.

## 2 MATERIALS AND METHODS

### 2.1 Data collection

The procedure for data collection was reviewed and preapproved by the Kansas State University Institutional Animal Care and Use Committee. Twenty‐four, client‐owned, healthy, male dogs presenting to Kansas State College of Veterinary Medicine for routine examination and exhibiting no signs of urinary tract disease (no reports of hematuria, pyuria, stranguria, polyuria, pollakiuria, incontinence, or micturition behavior abnormalities) were enrolled and examined on the same day as enrollment, with the written permission of their owners, over the course of 7, nonconsecutive days in a 1‐month period. Dogs were considered healthy by lack of owner‐reported health complaints, and lack of findings on physical examination that indicated urinary dysfunction or comorbidities of known consequence to urine production, micturition, or elimination behavior. Dogs of any breed, weight, age, or reproductive status were included. Urinalysis was not required for inclusion, and time since last micturition event was not controlled before examination. Each dog was placed in dorsal recumbency and a small quantity of transmissive ultrasound gel (Covidien, Dublin, Ireland) was applied to the caudoventral abdomen. Dogs were not shaved before evaluation. Sequential examinations using the BPP were performed by an experienced ultrasonographer (a third‐year veterinary diagnostic imaging resident with formal training in two‐dimensional [2D] ultrasonography) using both the “man” and the “child” settings of the device, then immediately thereafter by a novice ultrasonographer (an emergency veterinarian with >10 years clinical experience but no prior training in point‐of‐care or specialty ultrasonography). The order of the examinations by the 2 operators was not deliberately randomized, but varied from dog to dog; the second examiner was blinded to the results of the first examiner's scan. Both operators followed the prompts of the BPP until a bladder volume was reported, and the measure was repeated and recorded 3 times using each setting, by each operator, for a total of 12 BPP examinations per enrolled (3 by the experienced ultrasonographer using the man setting, 3 by the experienced ultrasonographer using the child setting, 3 by the novice ultrasonographer using the man setting, and 3 by the novice ultrasonographer using the child setting). Aseptic urinary catheterization was performed with a 3.5‐8.0 French red rubber catheter (Covidien), and the urine volume was quantified using a standard 500 mL polypropylene graduated cylinder. The catheter was advanced only far enough to freely allow urine flow by placing the tip of the catheter within the bladder, and urine was collected until flow ceased and did not resume with gentle repositioning. Traditional B‐mode ultrasonography using the BPP was assessed for complete voiding before catheter removal, defined as the inability to obtain additional urine with trace or minimal residual bladder contents assessed subjectively by the experienced ultrasonographer. Systematic assessment of bladder shape, size, position, and content was not performed, as in routine B‐mode ultrasonographic examination, though examiners were invited to comment and exclude dogs if incidental signs of disease were seen during the examination. No known complications occurred secondary to catheterization or related to the ultrasound procedure. Time of day and physical location of examination was not recorded or controlled.

### 2.2 Statistical analysis

Descriptive statistics, including mean volume of urine by operator, type of setting, and day of the test, are depicted in Table 1. Plots depicting percentage and absolute disagreement between averaged BPP examinations using the “man” setting and catheterized volume for each dog were produced in Microsoft Office Suite Excel 2013 (Microsoft, Redmond, Washington), as were descriptive statistics.

**TABLE 1:** Crude mean (SD) urine volume (mL) measured by BPP and by catheterization, segmented by operator, BPP method of measurement, and day of measurement

Method of measurement					
Day	Operator 1—Experienced	Operator 2—Novice			Actual
	Child	Man	Child	Man	Catheter
1	51.9 (40.3)	110.8 (32.9)	63.2 (47.1)	69.2 (54.2)	73.7 (64.5)
2	370.0 (134.7)	379.2 (171.2)	329.7 (157.4)	341.5 (103.2)	335.0 (205.1)
3	45.9 (19.3)	43.1 (16.4)	37.8 (20.5)	40.6 (19.6)	49.0 (16.0)
4	20.3 (10.2)	23.0 (0.0)	18.7 (3.5)	21.3 (2.5)	32.0 (.)
5	50.4 (16.2)	55.2 (14.7)	50.9 (12.8)	54.4 (13.1)	50.8 (23.3)
6	43.5 (32.5)	47.3 (32.9)	45.7 (36.7)	40.1 (30.7)	35.0 (22.7)
7	51.6 (53.3)	48.6 (51.8)	42.1 (36.5)	55.7 (57.2)	53.1 (54.1)
Mean	74.7 (103.5)	81.6 (110.9)	68.9 (95.2)	75.1 (94.9)	74.5 (98.9)
n	72	72	72	72	24

*Note:* Actual, volume recorded by catheter; n, number of observations.

Abbreviation: BPP, BladderScan Prime Plus.

Linear mixed models using a Gaussian distribution, identity link, restricted pseudo‐likelihood estimation and Newton‐Ridging optimization were fitted to evaluate the associations between different factors with mean volume of urine as detected by the BPP, using Proc Glimmix in SAS 9.3 (SAS Institute Inc, Cary, North Carolina). The outcome consisted of the logarithmic base 10 transformation of the mean volume of urine, recorded in milliliters. Independent variables consisted of the operator measuring the volume of urine (experienced or novice), method of measurement (“child” setting, “man” setting, or volume obtained by catheterization), the weight of the dog in kilograms and categorized into quartiles (≤5.5 kg, n = 6; 5.6‐11.94 kg, n = 6; 11.95‐24.9 kg, n = 5; >25.0 kg, n = 7), and the day the measurement was performed (categorical). Unconditional associations were tested in models including each of the predictor variables against the outcome, in individual models (Table 2). Multivariable models, including 2‐way interactions between each of the predictors of interest with the outcome, were also fitted (Table 3). Model assumptions and residuals were visually evaluated to identify normality and homoscedasticity. Model‐adjusted mean volumes, their corresponding 95% confidence intervals, and *P* values (α of .05) were reported.

**TABLE 2:** Univariable associations and relevant contrasts between operator, BPP method of measurement (setting), body weight, and day, with mean urine volume, and corresponding 95% CIs

Variable	Mean volume (mL)	Mean volume 95% CI (mL)	*P* value
Operator			.05*
Experienced (1)	40.4	25.8‐63.4	
Novice (2)	37.2	23.7‐58.3	
Method of measurement			<.001*
Child	36.3	23.1‐57.0	
Man	39.1	24.9‐61.4	
Actual	44.4	28.1‐70.0	
Body weight (kg)			.01*
≤5.5	18.5	8.4‐40.7	
5.6‐11.94	24.0	10.9‐52.9	
11.95‐24.9	114.0	48.0‐270.6	
≥25	49.1	24.7‐97.4	
Day of measurement			.21
1	31.1	8.5‐113.5	
2	323.1	66.3‐1574.7	
3	37.6	10.3‐137.0	
4	21.7	2.3‐203.8	
5	50.3	13.8‐183.3	
6	27.8	9.1‐85.3	
7	29.6	14.0‐62.6	

Abbreviations: BPP, BladderScan Prime Plus; CI, confidence interval.

**TABLE 3:** Relevant results of multivariable models (model‐adjusted means, 95% CIs, and *P* values) testing 2‐way interactions between BPP method of measurement, body weight, operator, and day of measurement, against mean volume of urine recorded

Variable	Mean	Mean 95% CI	*P* value
Method of measurement × Body weight			
*Method of measurement*			.002*
Child	37.4	25.1‐55.6	
Man	40.1	26.9‐59.6	
Actual	44.9	30.1‐67.1	
*Body weight (kg)*			.02*
≤5.5	19.6	8.9‐43.4	
5.6‐11.94	25.2	11.5‐55.7	
11.95‐24.9	110.0	46.1‐262.4	
≥25	50.3	25.3‐100.3	
*Two‐way pairs (setting—kg)*			.001*
Child: ≤5.5	17.4	7.8‐38.6	
Child: 5.6‐11.94	21.0	9.4‐46.5	
Child: 11.95‐24.9	117.1	48.9‐280.4	
Child: ≥25	45.7	22.8‐91.4	
Man: ≤5.5	17.5	7.9‐38.8	
Man: 5.6‐11.94	25.6	11.5‐56.8	
Man: 11.95‐24.9	118.4	49.4‐282.6	
Man: ≥25	48.8	24.4‐97.7	
Actual: ≤ 5.5	24.9	11.1‐55.9	
Actual: 5.6‐11.94	29.7	13.3‐66.7	
Actual: 11.95‐24.9	95.9	39.6‐232.3	
Actual: ≥25	57.2	28.3‐115.5	
Operator × Method of measurement			.23
Operator × Day			.64
Operator × Body weight			.46
Method of measurement × Day			.29

Significant contrasts for Method of measurement × Body weight interaction			
Weight (kg)	Relevant contrasts	Difference	*P* value
	Setting × Weight		
≤5.50	Actual versus child	7.5	.02*
	Actual versus man	7.4	.02*
≤ 5.50	Child versus man	−0.1	1.00
5.60‐11.94	Actual versus child	8.7	.03*
	Actual versus man	4.1	.92
5.60‐11.94	Child versus man	−4.6	.32
11.95‐24.9	Actual versus child	−21.2	.81
	Actual versus man	−22.5	.71
11.95‐24.9	Child versus man	−1.3	1.00
≥25.0	Actual versus child	11.5	.41
	Actual versus man	8.4	.84
≥25.0	Child versus man	−3.1	.99

*Note:* Relevant contrasts of method of measurement by body weight (kg) (the significant predictors) are appended.

Abbreviation: BPP, BladderScan Prime Plus; CI, confidence interval.

## 3 RESULTS

Mean bladder volumes recorded by the BPP using different settings and handled by different operators were compared to the measure of bladder volume by catheterization (Table 1). Operator, method of measurement (setting), and weight were significantly associated in univariable analyses with mean urine volume, whereas day of measurement was not (Table 2). On average, the experienced operator recorded volume readings that were 3.2 mL significantly (*P* = .05) higher than the novice operator (Table 2). In multivariable analysis, the association between the operator obtaining the measurements with the mean volume of urine did not depend on the method of measurement used (*P* = .23), the day the measurement was performed (*P* = .64), or the body weight of the dog (*P* = .46) (Table 3).

Using the “man” setting provided readings nearer to the catheterized urine measurements than the “child” setting, and this difference between settings was statistically significant (*P* < .001) (Table 2).

The mean volume of urine recorded by the instrument significantly (*P* = .01) varied by body weight, and the association between the type of setting with the mean volume of urine depended on the weight of the dog (*P* = .001) (Table 3). In dogs weighing ≤5.5 kg (n = 6), both the “man” (*P* = .02) and “child” (*P* = .02) settings produced significantly different urine volumes when compared to catheterized volumes. In the 5.6 to 11.94 kg group (n = 6), the “child” setting remained significantly different (*P* = .03), while the “man” setting was not (*P* = .92). In the remaining 11.95 to 24.9 kg group (n = 5) and ≥25.0 kg group (n = 7), neither the “man” setting nor the “child” setting produced readings significantly different from catheterized volume. However, the “child” setting in the 11.95 to 24.9 group showed a mean difference slightly closer to the catheterized volume mean than the “man” setting, a difference of −21.2 mL versus −22.5 mL, respectively. The lowest differences between catheterized measurements and either method of measurement (“man” or “child” setting) were found among lower weight (≤5.5 kg) animals. In contrast, and although not significantly different, the highest differences between measurements were observed among middle‐weight animals (11.95‐24.9 kg).

**FIGURE 1:**
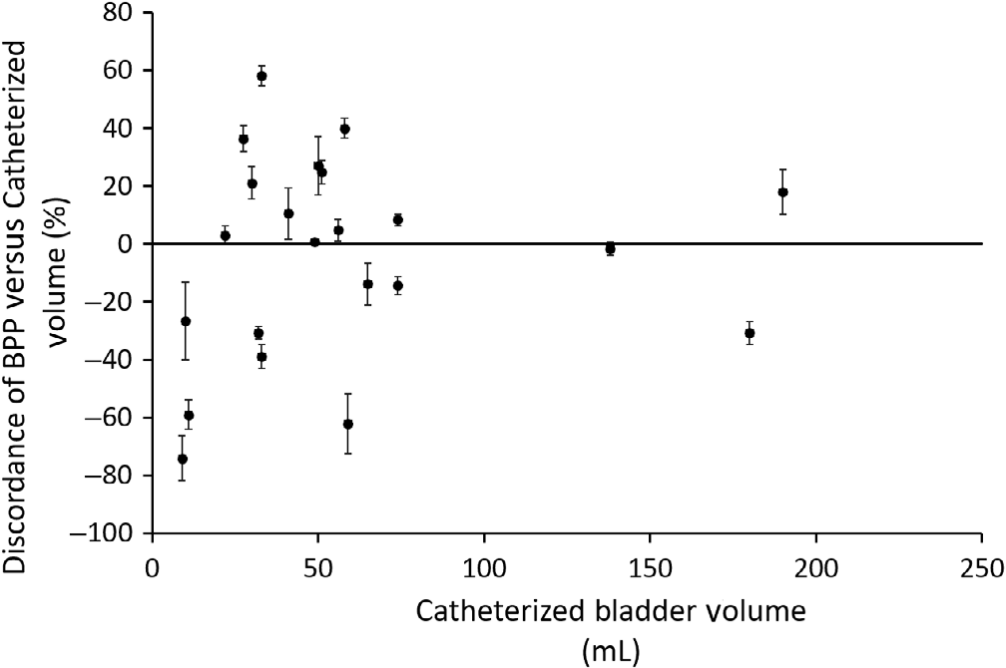
Plot of percent discordance between BPP man setting and catheterized bladder volume at varying bladder volumes. Each solid black point represents the average of 12 total measures completed for each dog, regardless of operator. Cath, catheterized volume; Man, volume recorded by BPP using “Man” setting; error bars, 95% confidence intervals. BPP, BladderScan Prime Plus

**FIGURE 2:**
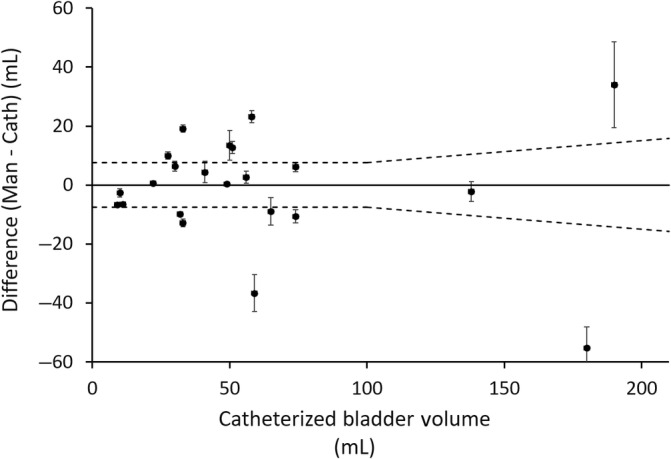
Agreement in absolute volume between BPP man setting and catheterized measures at variable bladder volumes. Each solid black point represents the average of12 total measures completed for each dog, regardless of operator. The hashed lines represent manufacturer claims of ±7.5 mL at volumes ≤100 mL and ±7.5% at volumes >100 mL. Cath, catheterized volume; Man, volume recorded by BPP using “Man” setting; error bars, 95% confidence intervals. BPP, BladderScan Prime Plus

Absolute and percentage disagreement between BPP results averaged across all examinations (6 total, using the “man” setting only, irrespective of operator) were calculated and compared to the mean of the BPP and catheterized result. (Figures 1 and 2) The differences in milliliters between BPP's “man” setting and catheterized volumes were normally distributed, and demonstrated a mean of −0.88 mL, median of 0.5 mL, SD of 19.0 mL, and maximal positive and negative disagreement of +23.2 mL to −55.3 mL. Percent disagreement between the mean BPP measurement and the catheterized volume demonstrated a mean of −4.53%, median of 1.86%, SD of 34.9%, and a maximal positive and negative disagreement of +58.1% to −74.1%. The percent discordance between catheterized volume and BPP was greatest at smaller bladder volumes (<100 mL), though absolute difference in milliliters varied most at large volumes. Eleven of 24 examined dogs had results using the “man” setting which fell within the tolerances claimed by the manufacturer for human examinations (±7.5% on volumes over 100 mL and ±7.5 mL on volumes less than 100 mL). By comparison, 10 of 24 of the examinations using the “child” setting met these same criteria.

The greatest catheterized urine volume was observed in dogs weighing 11.95 to 24.9 kg, followed by dogs >25 kg, and then <11.94 kg (Table 2). Mean urine volume did not significantly vary (*P* = .21) by the day the measurement was performed, nor did the day significantly affect the association between method of measurement and urine volume (*P* = .29) (Table 3).

During B‐mode use of the device, no incidental signs of subclinical urinary tract disease were detected in any of the reported dogs, including, but not limited to, echogenic content, mural thickening/masses, or suspended crystals.

## 4 DISCUSSION

Identification of urinary retention disorders can be challenging, particularly in cases of nonobstructed animals that appear to normally micturate, though void incompletely. For this reason, a validated method of quantifying bladder volume in dogs has been sought, as well as normal values for residual urine. Normal residual urine in dogs has historically been reported as 0.2 to 0.4 mL/kg, though wider variation has been documented, from 0.1 to 3.4 mL/kg.^9^, ^13^, ^14^, ^17^, ^18^ Estimation of bladder volume in dogs using 2D, B‐mode ultrasonography has also been established, utilizing linear length, width, and height measurements of the bladder (prolate ellipsoid method) or multiple cross sectional areas to approximate volume (multiple parasagittal sections method). Of defined methods, the prolate ellipsoid is often recommended by medical and veterinary authors because of its comparatively accurate results combined with ease of application.^9^, ^13^, ^14^, ^16^, ^17^, ^18^, ^19^, ^20^, ^21^, ^22^, ^23^, ^24^, ^25^ Despite the increasing availability of point‐of‐care ultrasonography, estimates of volume have not seen wide adoption in the experience of the authors, presumably due to several factors: they can present difficulty for untrained ultrasonographers, they add to examination time, and the relative value of the information they provide might not be perceived as superior to subjective assessments.

Use of the BPP resulted in accurate measurement of urine volume in the majority of dogs, depending on the selected setting and weight of the dog. This accuracy was demonstrated by lack of evidence of a significant difference between the catheterized measurement and mean volumes reported using the “man” setting in dogs >5.0 kg. However, the precision of the device could represent a potential shortcoming, with a SD for the disagreement between catheterized volume and the device's measurements of 34.9%, implying that ∼68% of examinations are expected to fall within a range of ±34.9% of the true urine volume. The clinical impact of this error will require additional study to elucidate. Dogs being evaluated for urinary retention disorders—some characterized by incomplete voiding, and others by total inability to micturate—might disproportionately distribute toward very large and somewhat small bladder volumes relative to their size. Furthermore, the threshold for diagnosis of urinary retention is a function of both weight and postvoiding bladder volume, with a third factor being the performance of the test selected, and a fourth factor being case selection for these examinations. As such, measuring the BPP's performance in a more narrowly defined group of cases and comparing its performance against other diagnostics available will be a necessary step to establish its clinical role.

A significant difference in recorded urine volumes was detected when comparing the mean BPP volumes reported by the 2 operators. The experienced operator had a significantly higher mean volume than the novice, with a magnitude difference of 3.2 mL between operator means. The operator means significantly varied from each other, with the experienced operator's mean nearer to the catheterized volume than the novice operator's, though neither significantly varied from the catheterized volume (Table 3, Method of Measurement × Operator). This suggests that either prior familiarity with ultrasound was of slight benefit to accuracy, that a nonskill‐related effect was responsible for this difference, or that this result was influenced by the small sample size. A different study design, including a larger cohort of experienced and inexperienced users sampling fewer dogs, would be preferred to detect skill‐related effects.

Device setting also influenced accuracy of volumetry, and this effect depended on the weight of the examined dog. In dogs weighing less than 5.0 kg, both the “man” and the “child” settings performed similarly, yet both significantly underestimated mean catheterized volumes by 7.4 mL and 7.5 mL, respectively. In all dogs weighing >5 kg, the mean difference in urine measurements between the device's “man” setting and the catheterized measurement, regardless of operator, was not significant, though in the 5.6 to 11.94 kg category, the “child” setting showed a significantly different result while the “man” setting remained nonsignificant. Overall, the “man” setting produced mean urine volumes nearer to the volume of urine collected by catheter (difference of −22.5 to +8.4 mL) for all weight classes save the 11.95 to 24.9 kg class, where the mean difference for the “child” setting was 0.3 mL closer to the catheterized measurement. This represented a magnitude difference that is subjectively of minor importance, suggesting that the “man” setting is superior in the majority of cases, and should be recommended in future applications.

Dogs in the 11.95 to 24.9 kg weight class had the largest mean volume of urine (catheterized), exceeding that of dogs ≥25 kg by a factor of 2.19. A prior study in which distensile volumes of fluid were instilled into the urinary bladder described a linear mL/kg relationship of weight and the state of distension produced.^26^ The unexpected inflection point in this result could be explained by a nonrandom distribution of samples with regards to state of bladder filling at the time of presentation, in combination with small sample size.

Three‐dimensional geometry acquisition for urinary bladder volumetry is theorized to offer advantages over previous 2D methods.^16^, ^23^ Derivation of volume from static 2D measurements relies on assumptions about uniformity of bladder shape, and necessitates application of empirically derived correction factors that might not apply to all veterinary species, nor do they account for changes in bladder conformation because of recumbency.^9^, ^13^, ^16^, ^18^, ^23^ The accuracy of calculated volumes relative to true bladder content varies, with varied evidence of as little as 3% to 4% discordance, or as much as 13%. This value could be influenced by the mathematical method selected, interoperator variability, intraoperator variability, or recumbency—for example, dorsal recumbency results in volume underestimation, whereas right lateral overestimates, presumably because of gravitational change in morphology of the bladder and divergence from the typical geometry.^9^, ^14^, ^18^

Manual tracing of organs boundaries in 3D imaging data sets can be time consuming, difficult, and impractical for general clinical use when compared to 2D boundary tracing or caliper measurement. Routine use of 3D ultrasonographic volumetry might necessitate algorithmic recognition of organ bounds, which will result in functions previously under direct control of ultrasonographers being relegated to the device's software; thus, the quality of the volumetric estimates using 3D‐ultrasound will be closely tied to the software and hardware layers of a selected device, and could be more instrument specific though less technique sensitive than prior methods. If such devices are to be utilized in research and clinical practice, validation studies are necessary, as manufacturers and models might differ in ways not immediately apparent to users. In the case of the BPP, the convolutional neural network relied upon by the device's software remains opaque from the user's perspective—that is, the weighted variables used to select boundaries and derive volume are not known, an example of the “black box” problem commonly cited in machine learning. It is therefore difficult to anticipate which, if any, anatomical structures or pathologies could “trick” the BPP's software without direct testing, because users are not privy to the decision‐making process of the algorithm. This uncertainty regarding the function of deep neural networks has been shown to be of consequence in medical image processing, where confounding variables (such as use of a portable radiograph unit) have been “accidentally” ranked highly by algorithms as indicators of disease, rather than condition‐specific imaging features.^27^ For this reason, real‐world application, stress testing, and development of feedback mechanisms for quality control of machine learning powered devices are essential.

Among the limitations of this study are a few drawbacks that could reduce the relevance of the study population. For example, breed and body condition were not recorded, and considering the wide spectrum of conformations encountered, results might not generalize to all dogs. Relatively few measurements of urine volume were greater than 100 mL, resulting in an unbalanced data set that reduced the strength of the conclusions for dogs with larger urine volumes. The decision to utilize only male dogs, selected for ease of catheterization, leaves undetermined whether the device is useful in female dogs.

The BPP's performance was not compared against traditional 2D‐ultrasound volumetry in this study, and thus any judgments about the superior method from the standpoint of accuracy cannot be made. Urine volume by catheterization was utilized as a gold standard for comparison, and catheterization is known to leave behind small volumes of urine, underestimating bladder volume.^14^ This confounder was minimized by ultrasonographic documentation of trace to minimal urine remaining in the bladder after catheterization; however, exact volumes were not accounted for, and this might influence results in the small study population. Intrarater agreement is an essential evaluative criterion for gauging the performance of medical devices which was not able to be sufficiently investigated in our study because of statistical limitations imposed by use of only 2 operators, with relatively few replicates performed on each dog. Finally, urinalyses were not required as an inclusion criterion, and therefore, subclinical urinary tract disease could have been present in dogs presumed to be normal. The relative impact of this variable on comparisons of volume was felt to be small.

Although interuser variability is present, our study provides early support toward use of the BPP for accurate, though imprecise quantification of bladder volume in dogs weighing greater than 5.5 kg. It is important to consider the precision of the device, however, before its use in clinical cases: for example, a small difference in absolute volume measured in a 5.5 kg dog could have a much greater impact on clinical decisions than the same volume difference in a 40 kg dog. In the research setting, the accuracy of the device could be acceptable when performing comparisons between larger populations, where repeated measures and use of central tendencies could minimize the impact of precision limitations.

## CONFLICT OF INTEREST DECLARATION

Funding was obtained independently from the Department of Clinical Science at Kansas State University. The BPP device was temporarily provided without charge to the investigators for the purposes of the study and for general application. The authors have no commercial interest in success of the tested product and were not compensated; there is no known conflict of interest for any author.

## OFF‐LABEL ANTIMICROBIAL DECLARATION

Authors declare no off‐label use of antimicrobials.

## INSTITUTIONAL ANIMAL CARE AND USE COMMITTEE (IACUC) OR OTHER APPROVAL DECLARATION

Kansas State University IACUC approval.

## HUMAN ETHICS APPROVAL DECLARATION

Authors declare human ethics approval was not needed for this study.
